# The roles of AKR1C1 and AKR1C2 in ethyl-3,4-dihydroxybenzoate induced esophageal squamous cell carcinoma cell death

**DOI:** 10.18632/oncotarget.7775

**Published:** 2016-02-27

**Authors:** Wei Li, Guixue Hou, Dianrong Zhou, Xiaomin Lou, Yang Xu, Siqi Liu, Xiaohang Zhao

**Affiliations:** ^1^ State Key Laboratory of Molecular Oncology, Cancer Hospital, Peking Union Medical College & Chinese Academy of Medical Sciences, Beijing, China; ^2^ CAS Key Laboratory of Genome Sciences and Information, Beijing Institute of Genomics, Chinese Academy of Sciences, Beijing, China; ^3^ University of Chinese Academy of Sciences, Beijing, China; ^4^ Proteomics Division, BGI-Shenzhen, Shenzhen, Guangdong, China; ^5^ Third School of Clinical Medicine, Southern Medical University, Guangzhou, China

**Keywords:** aldo-keto reductase 1C1/C2, ethyl-3, 4-dihydroxybenzoate, MRM, ESCC

## Abstract

The aldo-keto reductase (AKR) superfamily of enzymes is critical for the detoxification of drugs and toxins in the human body; these enzymes are involved not only in the development of drug resistance in cancer cells but also in the metabolism of polycyclic aromatic hydrocarbons. Here, we demonstrated that AKR1C1/C2 increased the metabolism of ethyl-3,4-dihydroxybenzoate (EDHB) in esophageal squamous cell carcinoma (ESCC) cells. Previous studies have shown that EDHB can effectively induce esophageal cancer cell autophagy and apoptosis, and the AKR1C family represents one set of highly expressed genes after EDHB treatment. To explore the cytotoxic effects of EDHB, esophageal cancer cells with higher (KYSE180) or lower (KYSE510) AKR1C expression levels were evaluated in this study. The proliferation of KYSE180 cells was inhibited more effectively than that of KYSE510 cells by EDHB treatment. Furthermore, the effective subunits of the AKR superfamily, AKR1C1/C2, were quantitatively identified using multiple reaction monitoring (MRM) assays. The sensitivity of esophageal cancer cells to EDHB was significantly attenuated by the siRNA knockdown of AKR1C1/C2. Moreover, the expression of autophagy inducers (Beclin, LC3II and BNIP3) and NDRG1 was significantly elevated in KYSE180 cells, but not in KYSE510 cells, after EDHB treatment. When autophagy was inhibited by 3-methyladenine, KYSE180 cells exhibited an increased sensitivity to EDHB, which may be a metabolic substrate of AKR1C1/C2. These results indicated that ESCC patients with high AKR1C1/C2 expression may be more sensitive to EDHB, and AKR1C1/C2 may facilitate EDHB-induced autophagy and apoptosis, thus providing potential guidance for the chemoprevention of ESCC.

## INTRODUCTION

Esophageal cancer is the eighth most common cancer and the sixth leading cause of cancer-related death worldwide [1]. Esophageal squamous cell carcinoma (ESCC) accounts for nearly 90% of esophageal cancer cases in Asian countries [2]. Currently, the precise etiology and pathogenesis of esophageal cancer are unclear, and there is a lack of effective methods for early diagnosis. As a consequence, the majority of esophageal cancer patients are diagnosed at intermediate and advanced stages; their therapeutic results are poor [3, 4], and the 5-year survival rate is no greater than 19% [5, 6]. The development and progression of esophageal cancer are multi-factorial, multi-stage progressive processes that result from the joint action of environmental and genetic factors. However, interventions that treat precancerous lesions could reverse the evolution of esophageal cancer. Phenolic compounds, such as ethyl-3,4-dihydroxybenzoate (EDHB), form a class of chemical anticancer agents that act on the Nrf2/ARE pathway. Such compounds are widely distributed in fruits and vegetables and show strong antioxidant effects.

EDHB is also known as protocatechuic acid ethyl ester. The monomer component of EDHB is present in a variety of plant species, mainly in the leaves and roots. EDHB was identified in studies on the composition of the peanut seed coat, wine, and tea leaves, and it exerts relatively low antioxidant activity [7–9]. Therefore, EDHB can be used as a fat and cream antioxidant, a food additive, and a pharmaceutical intermediate. EDHB is a substrate analog of prolyl hydroxylase and acts as a competitive inhibitor. It inhibits the ubiquitination/degradation of hypoxia inducible factor (HIF) and promotes the expression of downstream genes (e.g., erythropoietin and vascular endothelial growth factor), thus exerting critical biological effects [10]. EDHB also protects L6 myoblasts from hypoxia-induced oxidative damage [11] and inhibits collagen secretion from breast cancer cells, thus preventing tumor metastasis. This activity has been shown to reduce tumor fibrosis and metastasis in a mouse model of breast cancer, thereby providing a new potential treatment for breast cancer [12]. Previous research has also shown that EDHB inhibits esophageal cancer cell proliferation by increasing autophagy and apoptosis through NDRG1 and BNIP3 and markedly increases AKR1C1, AKR1C2, and AKR1C3 mRNA expression [13]. Therefore, it may be possible to identify new methods to inhibit esophageal cancer by investigating the effect of EDHB on esophageal cancer cells and its underlying molecular mechanism.

The members of the aldo-keto reductase (AKR) superfamily are monomeric cytoplasmic proteins with a molecular weight of 34–37 kDa. AKRs catalyze the reduction of aldehydes and ketones into the corresponding alcohol compounds, and reduced nicotinamide adenine dinucleotide phosphate (NADPH) serves as a coenzyme. The AKRs can be divided into 15 families, and the AKR1C subfamily includes four isoenzymes, AKR1C1–AKR1C4. These isoenzymes are capable of reducing 3-, 17-, and 20-ketone steroids *in vitro* [14] and of polycyclic aromatic hydrocarbons (PAHs) [15]. Additionally, AKR1C participates in the metabolism of numerous exogenous drugs and enhances the metabolism of certain endogenous and exogenous substrates. Many important drugs, such as doxorubicin, daunorubicin, haloperidol, and the novel anticancer drug oracin, are metabolized by carbonyl-reducing enzymes, including AKR1A1, AKR1B1, AKR1B10, AKR1C1, AKR1C2, and AKR1C3 [16]. However, EDHB is an exogenous substance with a similar structure to the metabolic substrates of AKR1C (i.e., PAHs). For this reason, we confirmed that EDHB is a metabolic substrate of AKR1C. The most recent study on this topic showed that prostate tumor samples with activated AKR1C1, an androgen-metabolizing enzyme, harbor high Sp1 and c-FLIP expression and low AKR1C1, ERβ and Sp3 expression. AKR1C1/ERβ activation induces apoptosis by downregulating c-FLIP expression, providing a new approach for the treatment of prostate cancer [17]. Our results showed that an esophageal cancer cell line overexpressing AKR1C was more sensitive to EDHB-induced cell death. However, in the AKR1C subfamily, AKR1C1/C2–AKR1C4 share a high degree of homology. In particular, the homology between AKR1C1 and AKR1C2 is 98%, with only seven different amino acids. To analyze which AKR1C protein increases EDHB-induced cell death in ESCC, we utilized a quantitative method, mTRAQ-based multiple reaction monitoring (MRM), to measure human AKR1C levels after EDHB treatment.

When combined with the newly released mTRAQ reagent, a non-isobaric variant of the iTRAQ tag that is available in two versions, the MRM approach enables the absolute quantification of peptides and proteins via isotope-dilution mass spectrometry. The method is conceptually similar to Western blotting but differs substantially in implementation, assay reliability, and the quality of the results. A Western blot essentially depends on antibody specificity. MRM is a better alternative for quantifying protein abundance using liquid chromatography (LC) or SDS-PAGE to separate proteins, especially when a suitable antibody is unavailable and highly precise quantification is necessary; Zhang et al. employed this method to quantitate the levels of AKR1A1, AKR1C1/C2, AKR1C3, AKR1C4, AKR1B1, and AKR1B10 in seven different cancer cell lines and to ascertain the absolute quantities of all proteins in each cell line [18–19]. In MRM, the mass spectrometer (MS) is set to monitor only specific mass/charge (*m/z*) values; as a consequence, the probability of detecting low levels of a peptide as part of a complex mixture is much higher [20]. Thus, MRM has been the method of choice for the quantification of similar proteins, especially when a suitable antibody is not available.

This study utilized an MRM assay to confirm that AKR1C1/C2 are involved in the EDHB-induced inhibition of esophageal cancer cell proliferation. EDHB was used as a substrate of AKR1C1/C2, and KYSE 180 cells overexpressing AKR1C1/C2 were more sensitive to EDHB, which potentially provides guidance for the use of EDHB for the treatment of esophageal cancer.

## RESULTS

### High levels of AKR1C enhanced the sensitivity of ESCC cells to EDHB

**Figure 1:**
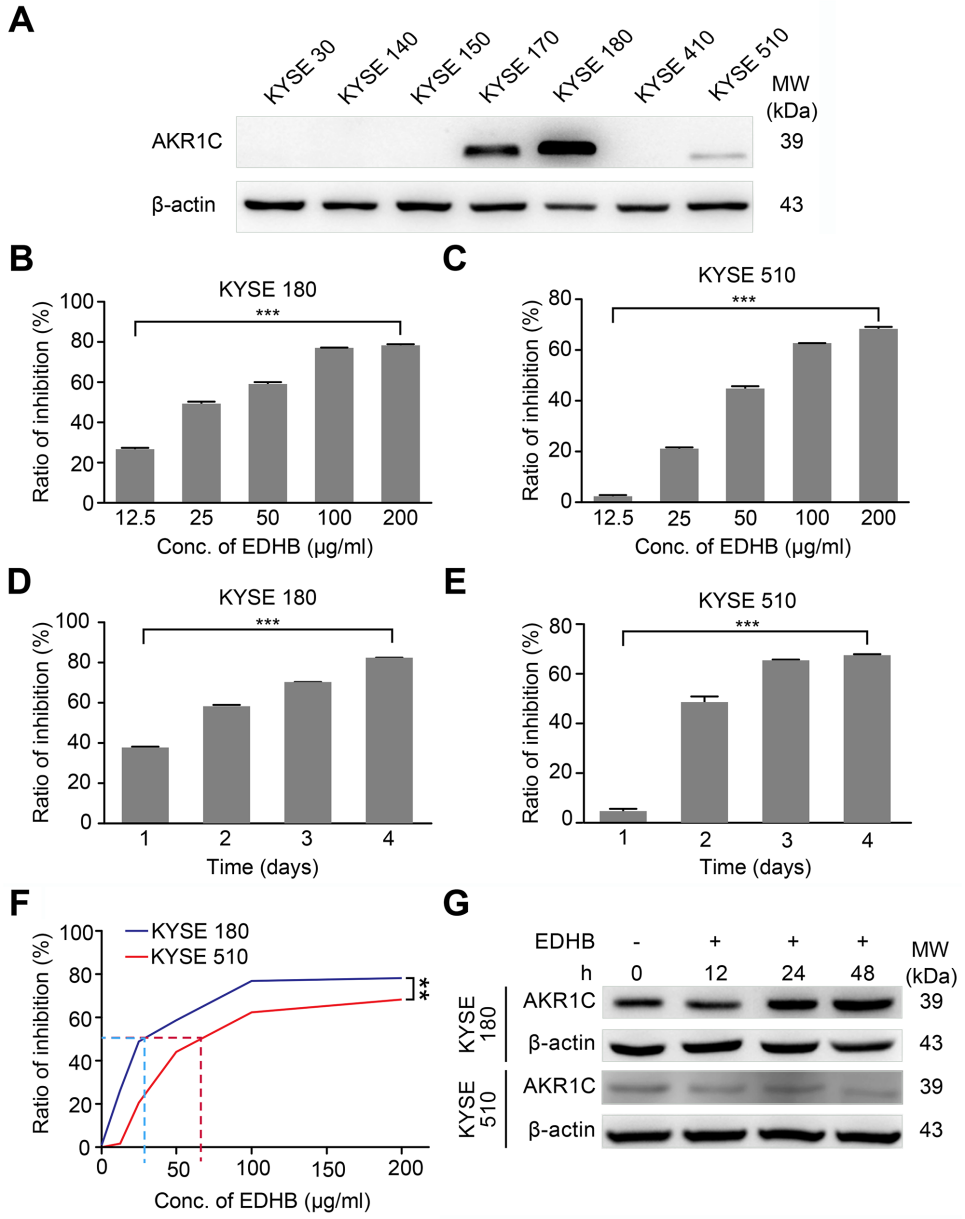
EDHB inhibited the proliferation of ESCC cells expressing high levels of AKR1C **A.** AKR1C expression in ESCC cell lines. β-Actin was used as an internal control. **B, C.** KYSE 180 cells (left, high AKR1C expression) and KYSE 510 cells (right, low AKR1C expression) were treated with various concentrations of EDHB for 2 days, and the ratio of the inhibition of cell proliferation was determined using WST-8 assays. **D, E.** KYSE 180 cells (left) and KYSE 510 cells (right) were treated with 40 or 80 μg/ml EDHB, respectively, for different periods of time, and the ratio of the inhibition of cell proliferation was determined using WST-8 assays. **F.** KYSE 180 and KYSE 510 cells were treated with the same concentration of EDHB for 2 days, and the inhibition of cell proliferation was determined using WST-8 assays. **G.** AKR1C expression levels: Western blotting of AKR1C was performed using 20 μg of total protein lysate from EDHB-treated KYSE 180 (upper panel) or KYSE 510 (lower panel) ESCC cells. Conc., concentration; ***, *P* < 0.001; **, *P* < 0.01.

To analyze AKR1C expression in different ESCC cells, total protein was extracted from KYSE 30, KYSE 140, KYSE 150, KYSE 170, KYSE 180, KYSE 410 and KYSE 510 cells. Western blot analysis showed that AKR1C was expressed at higher (KYSE 180) or lower (KYSE 510) levels in certain ESCC cell lines (Figure 1A). KYSE 180 and KYSE 510 cells were then treated with varying concentrations of EDHB, and the effect of AKR1C on the EDHB-induced inhibition of ESCC cell proliferation was evaluated. Two days after treatment, EDHB inhibited ESCC (KYSE 180 and KYSE 510) cell proliferation by more than 50% compared with the effect in control cells. The EDHB-induced inhibition of the proliferation of both cell lines was dose dependent; an increasing EDHB concentration evoked a greater reduction in cell growth (Figures 1B and 1C). Additionally, EDHB inhibited ESCC cell proliferation in a time-dependent manner (Figures 1D and 1E). Based on statistical calculations, the estimated IC_50_ of EDHB was 32.1 μg/ml in KYSE 180 cells and 74.8 μg/ml in KYSE 510 cells (Figure 1F). Thus, EDHB inhibited the proliferation of ESCC cells with either high or low AKR1C expression. To examine the role of AKR1C in the EDHB-induced inhibition of ESCC cell proliferation, KYSE 180 and KYSE 510 cells were treated with the same concentration of EDHB for 48 h; the inhibition of cell growth was greater in KYSE 180 cells than in KYSE 510 cells (Figure 1F). Western blot analysis revealed that AKR1C levels increased in KYSE 180 cells after prolonged treatment with 40 μg/ml EDHB but remained unchanged in KYSE 510 cells treated with 80 μg/ml EDHB (Figure 1G). These results showed that KYSE 180 cells, which express high AKR1C levels after EDHB treatment, were clearly more sensitive to EDHB and that AKR1C expression increased with prolonged EDHB treatment.

### Increased AKR1C1/C2 expression corresponded with cell growth inhibition by EDHB as determined by mTRAQ/MRM quantitative analysis

**Figure 2:**
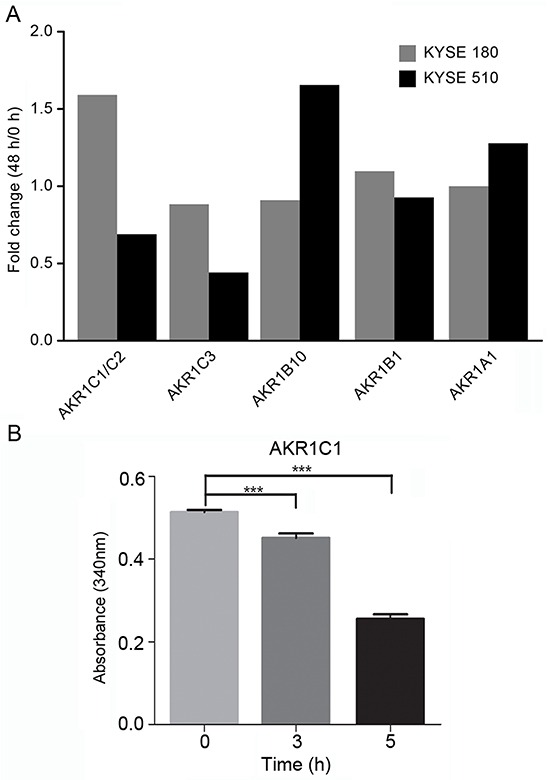
AKR1 quantification in ESCC cell lines (KYSE 180 and KYSE 510) via MRM assay **A.** Comparison of AKR abundance in KYSE 180 and KYSE 510 cells treated with EDHB for 48 h based on MRM quantification. The MRM signals were acquired for mTRAQ-labeled KYSE 180 and KYSE 510 cell lysates, and the corresponding AKR abundance was calculated; the data are shown in the bar graph. **B.** We investigated EDHB as a substrate of NADPH/NADH-dependent AKR1C1 reduction by detecting the change in the absorbance of the pyridine nucleotide at 340 nm in a 1-ml volume.

The human AKR superfamily contains aldose reductases, aldehyde reductases, hydroxysteroid dehydro- genases, and steroid 5β-reductases. The AKR1C subfamily includes four isoenzymes, AKR1C1–AKR1C4, that share a high degree of similarity. To determine which AKR1 isoenzyme was elevated in ESCC cells after EDHB treatment, KYSE 180 and KYSE 510 cells were treated with EDHB for 48 h, and the protein levels of AKR1C1/C2, AKR1C3, AKR1B1, AKR1A1, and AKR1B10 were then quantitatively analyzed by MRM. This method is more accurate than Western blotting for protein quantification. AKR1C1/C2 expression was elevated in KYSE 180 cells, whereas there was no obvious change in KYSE 510 cells after EDHB treatment. Furthermore, there was no significant change in the amount of AKR1C3, AKR1B1, AKR1A1, or AKR1B10 in KYSE 180 cells after EDHB treatment (Figure 2A). These results indicated that KYSE 180 cells overexpressed AKR1C1/C2 after EDHB treatment. Due to the structural similarities between AKR1C substrates and EDHB, we hypothesized that EDHB may be a substrate of AKR1C1. Therefore, NADPH/NADP-dependent AKR1C1 reduction was investigated using EDHB as the substrate; the absorbance at 340 nm decreased over time. Therefore, EDHB is a substrate of AKR1C1 (Figure 2B).

### Genetic knockdown of AKR1C1/C2 attenuated the sensitivity of ESCC cells to EDHB

**Figure 3:**
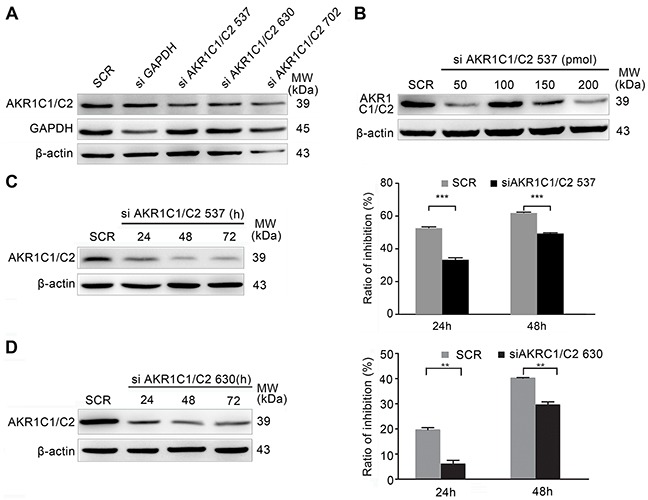
EDHB sensitivity of AKR1C1/C2-depleted KYSE 180 cells **A.** The efficiency of AKR1C1/C2 knockdown using different AKR1C1/C2 siRNAs was determined by Western blot analysis. **B.** KYSE 180 cells were transfected with various concentrations of siAKR1C1/C2 537 using Lipofectamine 2000. Knockdown efficiency was determined by Western blot analysis. **C, D.** A total of 50 pmol of siAKR1C1/C2 537 (C) or siAKR1C1/C2 630 (D) was transfected using Lipofectamine 2000 for 3 days. During this time, KYSE 180 cells were treated with 40 μg/ml EDHB. The MTT assay was performed after EDHB treatment for 1 or 2 days, and the ratio of inhibition was determined relative to untreated cells (mean + S.E.M. for triplicate samples).

To further ascertain that AKR1C1/C2 increased the EDHB-induced inhibition of esophageal cancer cell proliferation, AKR1C1/C2 were depleted using different siRNAs, and knockdown efficiency was determined by Western blot (Figure 3A). The effects of different concentrations of siAKR1C1/C2 537 were determined by Western blotting (Figure 3B). A total of 50 pmol of siAKR1C1/C2 537 or siAKR1C1/C2 630 was selected based on the most efficient AKR1C1/C2 knockdown. After treatment with siAKR1C1/C2 537 or siAKR1C1/C2 630, the sensitivity of ESCC cells to EDHB was investigated; the EDHB-induced inhibition of cell proliferation was significantly reduced by different siAKR1C1/C2 constructs (Figures 3C and 3D), indicating that AKR1C1/C2 promoted the EDHB-induced inhibition of ESCC cell proliferation.

### AKR1C1/C2 increased EDHB-induced esophageal cancer cell apoptosis

**Figure 4:**
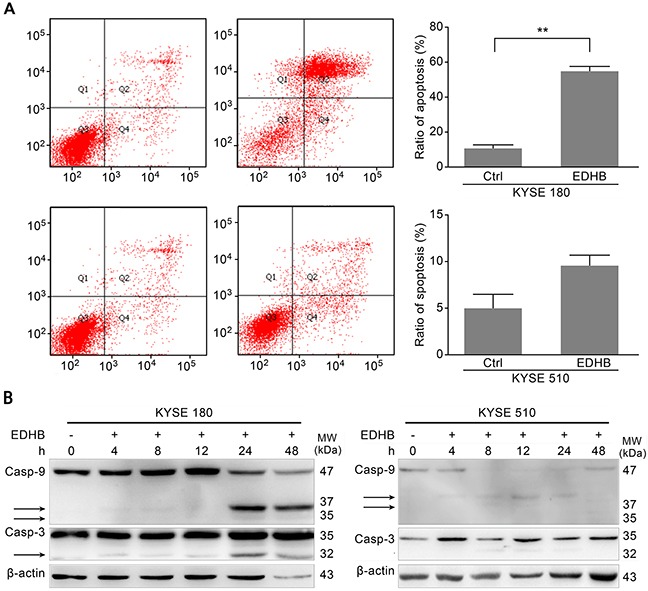
EDHB differentially induced apoptosis in ESCC cells with different AKR1C1/C2 expression levels **A.** KYSE 180 and KYSE 510 cells were treated with 40 or 80 μg/ml EDHB, respectively, for 2 days. The cells were then stained with Annexin-V and PI, and the percentage of apoptotic cells was determined by flow cytometry. The data are shown graphically in the left panels. Ctrl, control; **, *P* < 0.01. **B.** KYSE 180 (left) and KYSE 510 (right) cells were treated with EDHB for 0, 4, 8, 12, 24, or 48 h, and caspase-3 and caspase-9 were analyzed by Western blot. h, hours.

Overexpression of AKR1C1/C2 increased the EDHB-induced inhibition of esophageal cancer cell proliferation. To further investigate the corresponding mechanism, Annexin V/propidium iodide (PI) staining was performed to detect apoptotic KYSE 180 and KYSE 510 cells after EDHB treatment for 48 h. The proportion of late apoptotic EDHB-treated KYSE 180 cells was significantly higher than that of EDHB-treated KYSE 510 cells (57.5% *vs.* 9.2%). There was a significant difference before and after EDHB treatment in KYSE 180 cells, but not in KYSE 510 cells (*P* < 0.01; Figure 4A). The proportion of apoptotic KYSE 180 cells was significantly higher than that of KYSE 510 cells after EDHB treatment (*P* < 0.01). To further evaluate KYSE 180 cell apoptosis, Western blotting was performed to examine caspase-3 and caspase-9 activation after EDHB treatment. The activated fragments of caspase-3 and caspase-9 were detected in KYSE 180 cells at 24 h after EDHB treatment, indicating apoptosis (Figure 4B). Caspase-3 and caspase-9 expression was lower in KYSE 510 cells than in KYSE 180 cells. Weak expression of cleaved caspase-9 was detected after an 8 h treatment with EDHB (Figure 4B). These results were consistent with the flow cytometry analysis, which showed a reduced proportion of apoptotic KYSE 510 cells after EDHB treatment.

### AKR1C1/C2 promoted EDHB-induced esophageal cancer cell autophagy

**Figure 5:**
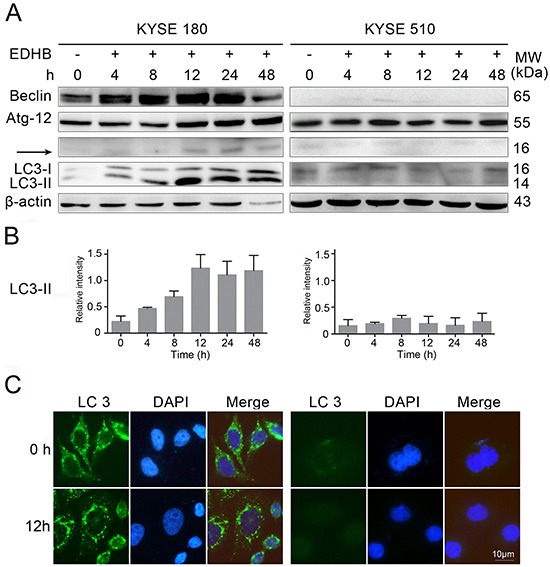
EDHB differentially induced autophagy in ESCC cells with different AKR1C1/C2 expression levels **A.** Beclin, Atg12, and LC3 protein levels were determined by Western blot using 20 μg/lane of protein lysate from ESCC cells (KYSE 180 and KYSE 510) treated with EDHB for 0, 4, 8, 12, 24, or 48 h. **B.** LC3 protein levels were quantitated by gray-slot scanning after EDHB treatment for 0, 4, 8, 12, 24, or 48 h; three biological replicates were analyzed. **C.** KYSE 180 and KYSE 510 cells were treated for 12 h with 40 or 80 μg/ml EDHB, respectively, and LC3 aggregation was visualized by anti-LC3 antibody staining and fluorescence microscopy. DAPI was used to stain the cell nuclei. Scale bar, 10 μm. KYSE 180 cells, left; KYSE 510 cells, right.

EDHB induced apoptosis in ESCC cells expressing high levels of AKR1C1/C2. To determine whether AKR1C1/C2 promotes EDHB-induced autophagy in ESCC cells, autophagy-related proteins were analyzed in EDHB-treated KYSE 180 and KYSE 510 cells. The levels of the autophagy markers Beclin, Atg12, and LC3-II were elevated in KYSE 180 cells at 12 h after EDHB treatment, whereas no changes in these markers were observed in EDHB-treated KYSE 510 cells (Figure 5A). LC3 expression was analyzed by gray-slot scanning; LC3-II levels increased in KYSE 180 cells with prolonged processing time, but there was very low expression in KYSE 510 cells (Figure 5B). Additionally, immunofluorescence revealed the presence of LC3 puncta and high LC3 expression in KYSE 180 cells at 12 h after EDHB treatment, but there were no significant changes in KYSE 510 cells after EDHB treatment at the same exposure intensity (Figure 5C). Taken together, these results suggested that AKR1C1/C2 promoted EDHB-induced ESCC cell autophagy.

### EDHB-induced inhibition of esophageal cancer cell growth was augmented by co-treatment with an autophagy inhibitor

**Figure 6:**
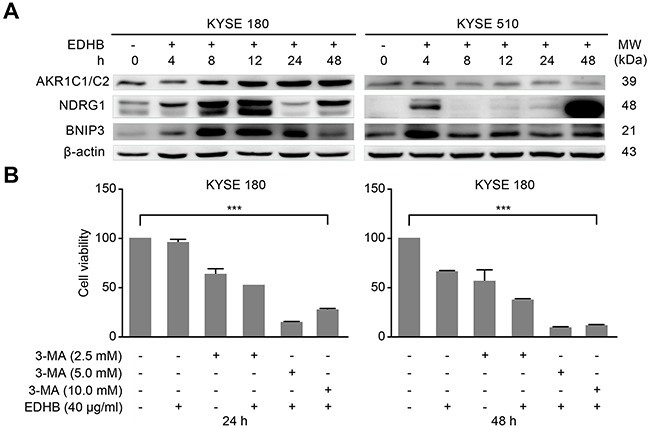
3-Methyladenine enhanced the EDHB-mediated inhibition of ESCC cells **A.** ESCC cells were treated with 40 μg/ml (KYSE 180) or 80 μg/ml (KYSE 510) EDHB for 0, 4, 8, 12, 24, or 48 h, and the expression of AKR1C1/C2, NDRG1, and BNIP3 was analyzed by Western blot. h, hours. Left, KYSE 180 cells; right, KYSE 510 cells. **B.** KYSE 180 cells were co-treated with EDHB (40 μg/ml) and various concentrations of 3-MA for 24 h (left) or 48 h (right), and cell viability was determined by WST-8 assays.

Previous studies have shown that EDHB induces apoptosis and autophagy in KYSE 170 cells by upregulating the expression of BNIP3 and NDRG1 [13]. To examine whether AKR1C1/C2 expression promotes cellular apoptosis and autophagy induced by BNIP3 and NDRG1, Western blotting was conducted; after EDHB treatment, BNIP3 and NDRG1 expression increased with longer EDHB treatment times in KYSE 180 cells but increased only after treatment for 4 h with EDHB in KYSE 510 cells, which was consistent with the results of the apoptosis analysis. These findings indicated that AKR1C1/C2 facilitated the induction of apoptosis and autophagy by BNIP3 and NDRG1 in esophageal cancer cells after EDHB treatment (Figure 6A). AKR1C inhibitors or other similar drugs are considered promising agents for adjuvant therapy, with the ability to prolong the anticancer efficacy of chemotherapy by two-fold [21]. In this study, we found that the EDHB-induced inhibition of ESCC cells was increased by co-treatment with varying concentrations of 3-methyladenine (3-MA), an autophagy inhibitor (Figure 6B).

Thus, increased levels of AKR1C1/C2 enhanced the sensitivity of ESCC cells to EDHB. AKR1C1/C2 facilitated the induction of early autophagy and late apoptosis by BNIP3 and NDRG1 in ESCC cells after EDHB treatment, thereby promoting the EDHB-induced inhibition of esophageal cancer cell proliferation. Additionally, AKR1C1/C2 may act as a metabolic enzyme with EDHB as a substrate to inhibit cell proliferation. Finally, the EDHB-induced inhibition of ESCC cells was increased by co-treatment with an autophagy inhibitor.

## DISCUSSION

Esophageal carcinoma has a poor prognosis and highly malignant behavior [5]. The majority of esophageal cancer patients are diagnosed at intermediate and advanced stages and respond poorly to chemotherapy [6]. Concurrent chemo/radiotherapy is currently the standard of care for the nonsurgical management of advanced esophageal cancer. For these reasons, additional adjuvant therapeutics are needed for esophageal carcinoma treatment.

EDHB is a phenolic compound that is mainly derived from plant leaves and roots and can be obtained through organic solvent extraction, which yields a compound with anti-tumor, anti-inflammatory, and analgesic effects. EDHB has been identified in peanut seed coat, wine, and tea leaves, and it exhibits relatively low antioxidant activity [7–9]. The benzene ring of EDHB contains two adjacent phenolic hydroxyl groups that structurally resemble the two adjacent phenolic hydroxyl groups in PAHs [15]. Recent research found that EDHB is a monomer substrate analog of α-ketoglutarate that inhibits collagen prolyl hydroxylase and prevents collagen synthesis [21–22]. EDHB inhibits collagen secretion by breast cancer cells and thus prevents tumor metastasis [12]. As a prolyl hydroxylase inhibitor, EDHB completely blocks ascorbic acid-induced osteoblast differentiation [23], prevents corneal epithelial wound healing [24], and treats ischemic diseases [25]. Additionally, Han et al. observed cell growth inhibition, cell cycle arrest, and a reduction in the mitochondrial membrane potential in ESCC cells after EDHB treatment; these effects were accompanied by late apoptosis induced by NDRG1 and early autophagy induced by BNIP3. Furthermore, cDNA microarray analysis revealed that the expression of AKR1C subfamily members is elevated in ESCC cells after EDHB treatment [13].

In this study, we quantitatively analyzed the key members of the human AKR superfamily involved in the EDHB-induced inhibition of cell proliferation and confirmed their function during this process. Cell proliferation was inhibited by EDHB in ESCC cells with either higher (KYSE 180) or lower (KYSE 510) AKR1C expression, and this inhibition occurred in a dose- and time-dependent manner. The results indicated that AKR1C overexpression increased the sensitivity of ESCC cells to EDHB. The AKR superfamily includes 15 families [26–27]; the human-derived AKRs include families 1, 6, and 7 [28–29]. Based on evolutionary conservation, members with an amino acid identity greater than 60% are defined as one subfamily [30]. In the AKR1C subfamily, AKR1C1/C2–AKR1C4 share a high degree of homology. In particular, the homology between AKR1C1 and AKR1C2 is 98%, with only seven different amino acids. To analyze which unique AKR1C protein was elevated after EDHB treatment, the mTRAQ/MRM method was used to quantify the human AKR family members for which no specific antibodies are available. The results revealed that AKR1C1/C2 expression was elevated in KYSE 180 cells at 48 h after EDHB treatment. Based on this result, we confirmed that AKR1C1/C2 are involved in the EDHB-induced inhibition of ESCC cell proliferation.

Additionally, AKRs are metabolic enzymes that use NADPH as a coenzyme to reduce carbonyl groups into monohydric and dihydric alcohols. AKRs have a wide range of substrates, including aldehydes, ketones, monosaccharides, steroids, prostaglandins, bile acid precursors, and chemical carcinogens and their metabolites [31–33]. AKR1C members are mainly involved in steroid hormone metabolism (AKR1C1/C2–3), prostate-related hormone metabolism (AKR1C3), and bile acid metabolism (AKR1C4). Moreover, AKR1C subfamily members are critical enzymes for the detoxification of drugs and toxins in the body. AKR1C1/C2 and AKR1C4 decreased the cytotoxicity of aldehydes and cisplatin in the HCT15 colon cancer cell line [34]. AKR1C1/C2–AKR1C3 can metabolize progesterone and tobacco carcinogens [35–36]. It was previously found that treatment of different oral squamous cells with cigarette smoke condensate (CSC) can increase the expression of AKR1C1/C2, AKR1C3, and AKR1B10 [37]. PAHs, which are AKR1 substrates, are the main type of chemical carcinogen in tobacco, and they exhibit mutagenic and carcinogenic effects [38–41]. EDHB is an exogenous substance with a structure similar to that of metabolic substrates of AKR1C (i.e., PAHs) [15]. We analyzed AKR1C1 activity and confirmed that EDHB acts as a metabolic substrate of AKR1C1 and increases the sensitive of ESCC cells to EDHB. To further demonstrate the role of AKR1C1/C2 in the EDHB-induced inhibition of ESCC cell proliferation, AKR1C1/C2 were depleted using different siRNAs, which significantly attenuated the inhibitory effect. Taken together, these results indicated that AKR1C1/C2 may be involved in the cellular metabolism of EDHB and increase the sensitivity of ESCC cells to EDHB.

Han et al. previously observed late apoptosis induced by NDRG1 and early autophagy induced by BNIP3 in ESCC cells after EDHB treatment [13]. Autophagy is a lysosomal degradation pathway that degrades proteins and cytoplasmic organelles by activating a series of autophagy-associated genes (ATGs) and the formation of autophagosomes. During autophagosome formation, the cytoplasmic form of LC3 (LC3-I) binds to phosphatidylethanolamine (PE) to form the LC3-PE complex (LC3-II), which is then recruited to the autophagosome membrane. Therefore, conversion from LC3-1 to LC3-II is a unique characteristic of autophagy. In evaluating the function of AKR1C1/C2 in EDHB-induced ESCC cell death, we showed that LC3-II and Beclin levels were elevated in KYSE 180 cells, which express high AKR1C1/C2 levels, at 12 h after EDHB treatment. Immunofluorescence revealed the presence of LC3 puncta in KYSE 180 cells at 12 h after EDHB treatment. This phenomenon was absent in KYSE 510 cells, which exhibit low AKR1C1/C2 expression, under the same conditions. In the endogenous apoptotic pathway, caspase-9 is activated and further activates caspase-3, thereby inducing apoptosis. Flow cytometry analysis showed that the proportion of apoptotic KYSE 180 cells was higher than that of KYSE 510 cells after treatment with the same concentration of EDHB. Western blot analysis revealed that caspase-3 and caspase-9 were activated in KYSE 180 cells at 48 h after EDHB treatment, whereas the expression of these proteins was very low in KYSE 510 cells. After treatment for 8 h with EDHB, weak cleaved caspase-9 expression was detected. These results were consistent with the flow cytometry analysis that showed a smaller proportion of apoptotic KYSE 510 cells after treatment with EDHB. Thus, EDHB treatment induced early autophagy and late apoptosis in KYSE 180 cells (high AKR1C1/C2) but not in KYSE 510 cells (low AKR1C1/C2). Meanwhile, NDRG1 and BNIP3 expression increased in KYSE 180 cells with prolonged EDHB treatment, whereas the expression of BNIP3 and NDRG1 increased at only the 4 h timepoint in KYSE 510 cells, and there were no time-dependent changes, which may be consistent with the apoptosis analysis. Thus, this study demonstrated that AKR1C1/C2 contributed to late apoptosis induced by NDRG1 and early autophagy induced by BNIP3 in KYSE 180 ESCC cells after EDHB treatment.

Another small inhibitor has emerged as a promising therapeutic for the treatment of a range of genetically defined hematologic malignancies. This inhibitor can be potentially utilized to treat a broader spectrum of human cancers that bear similar genetic and epigenetic characteristics [42]. Our results showed that the inhibition of cell proliferation by EDHB was more pronounced when early autophagy was inhibited with 3-MA. This observation provides an important contribution to the field of precision medicine by clarifying appropriate treatments for specific disease subtypes.

In summary, our observations indicated that AKR1C1/C2 promoted the inhibition of cell proliferation through either EDHB metabolism or BNIP3 and NDRG1 expression and induced early autophagy and late apoptosis in esophageal cancer cells. EDHB is an extracellular substance that structurally resembles PAHs. AKR1C1/C2 levels increased in KYSE 180 cells, which were more sensitive to autophagy and apoptosis after EDHB treatment. These results indicated that esophageal cancer patients with high AKR1C1/C2 expression may be more susceptible to EDHB, thus providing potential guidance for the chemoprevention and clinical treatment of ESCC.

## MATERIALS AND METHODS

### Reagents

EDHB was purchased from Sigma-Aldrich (St. Louis, MO) and dissolved in ethanol for biochemical assays. Anti-AKR1C antibodies were obtained from Santa Cruz Biotechnology (Santa Cruz, CA). Anti-caspase-3 and anti-caspase-9 antibodies were procured from Enzo Life Sciences (NY). Anti-LC3A/B and anti-Beclin antibodies were purchased from Cell Signaling Technology (Boston, MA). Anti-β-actin antibodies were obtained from Sigma-Aldrich (St. Louis, MO). Anti-BNIP3 and anti-NDRG1 antibodies were purchased from Abcam (Abcam, MA).

### Cell lines and cell culture

The human ESCC cell lines KYSE 180 and KYSE 510 were a generous gift from Dr. Y Shimada (Kyoto University, Japan). These cells were cultured in complete RPMI 1640 medium supplemented with 10% fetal bovine serum (FBS), penicillin (100 U/ml), and streptomycin (100 mg/ml) at 37°C in a humidified incubator containing 5% CO_2_.

### Western blot analysis

Protein was extracted from KYSE 180 and KYSE 510 cells after treatment with 40 or 80 μg/ml EDHB, respectively. Cells were washed twice with phosphate-buffered saline (PBS), lysed with RIPA buffer (50 mM Tris-HCl, pH 8.0, with 150 mM NaCl, 1.0% IGEPAL CA-630 (NP-40), 0.5% sodium deoxycholate, and 0.1% SDS) containing a protease inhibitor (500 mM phenylmethylsulfonyl fluoride) for 30 min, and sonicated. The cell extract was prepared by centrifugation (12,000 rpm for 15 min). Subsequently, the protein samples (25 μg) were electrophoresed on 10% SDS-PAGE gels and transferred to Millipore polyvinylidene difluoride membranes by electroblotting. The membranes were blocked for 3 h with 5% (w/v) nonfat dry milk in PBS and incubated with primary antibodies at 4°C overnight. Western blot analysis was performed using the following antibodies: anti-AKR1C, anti-caspase-9, anti-caspase-3, anti-NDRG1, anti-BNIP3, anti-Beclin, and anti-LC3. Proteins were detected with horseradish peroxidase-conjugated secondary antibodies and visualized with Renaissance Plus Reagent (Life Technologies, Grand Island, NY).

### Cell proliferation assay

WST-8 assays for examining cell proliferation were performed using a Cell Counting Kit-8 (CCK-8) (Dojindo, Japan). Cells in the logarithmic growth phase were plated in 96-well plates at a density of 1×10^4^ cells/well. The cells were treated with varying concentrations (0, 12.5, 25, 50, 100, or 200 μg/ml) of EDHB for 24, 48, 72, or 96 h. Untreated cells were used as a negative control. Culture medium (100 μl) was added to each well. The cells were then incubated with 10 μl/well of WST-8 in the culture medium for 3 h. Absorbance values were detected at 450 nm using an enzyme-linked immunosorbent assay (ELISA) reader. The IC_50_ value (50% inhibition of cell growth) was calculated graphically. Each column represents the average of at least two independent experiments performed in triplicate.

### AKR1C quantification via MRM assay

The cells (KYSE 180 and KYSE 510) were sonicated in lysis buffer (4% SDS, 7 M urea, 10 mM EDTA, 10 mM PMSF, 10 mM DTT, and 0.1 M Tris-HCl, pH 7.6). After sonication, the cell lysates were centrifuged at 25,000 g, and the resulting supernatants were reduced with 10 mM DTT and alkylated with 55 mM iodoacetamide. The treated proteins were digested with trypsin according to the FASP protocol as described by Mann [43]. Total protein content was measured using the Bradford assay and confirmed by SDS-PAGE. The MRM assay was performed on a QTRAP5500 MS (AB SCIEX, Framingham, MA) equipped with a nanoACQUITY UPLC system (Waters, Milford, MA) with a nanocapillary column (ID 75 μm × 20 cm, 1.7 μm particles, 100 Å apertures, Waters, Milford, MA). The peptides prepared from cell lysates were eluted with a non-linear gradient program at 300 nl/min. The mobile phases consisted of solvent A, 2% acetonitrile with 0.1% aqueous formic acid, and solvent B, 98% acetonitrile with 0.1% formic acid. Peptides were separated in and eluted with a gradient of 5-35% solvent B for 40 min followed by 35%-90% solvent B for 5 min. The MS parameters for all the MRM experiments were as follows: ion spray voltage (IS), 2500 V; curtain gas (CUR), 35.00; ion source gas 1 (GS1), 20.00; collision gas (CAD), high; interface heater temperature (IHT), 150; declustering potential (DP), 100.00; entrance potential (EP), 10.00; and Q1 and Q3, unit resolution. The spectra of 15 β-gal peptides were assessed daily as QC for the LC-MS system. The MRM methods and raw data were processed using Skyline [44], an open-source software (http://skyline.maccosslab.org/).

### Cloning, production, and purification of recombinant AKR1C1 proteins

DNA encoding full-length AKR1C1 (accession number NM_001353) was obtained from OriGene and cloned into pEX-N-His. Plasmids were transformed into *Escherichia coli* BL21 (DE3) cells to produce NH2-terminally His6-tagged recombinant proteins. Protein expression occurred without the need for induction with isopropyl-β-D-1-thiogalactopyranoside. Bacterial cultures were pelleted by centrifugation (3,800 × *g*, 15 min), and the cells were disrupted by resuspension in Bug-Buster reagent (Novagen) containing Benzonase or DNase (Novagen) at 10 ml lysis reagent/400 ml original culture. After incubation at room temperature (RT) with shaking (150 rpm) for 25–30 min, the cell debris was pelleted by centrifugation (48,000 × *g*, 30 min, 4°C). The cleared lysate was extracted using HiTrap Benzamidine FF (high sub) from GE Healthcare (Sweden).

### AKR1C1 enzymatic assays of EDHB reduction

Human AKR1C1 enzyme assays were performed at 35°C using 15 μg of recombinant protein in 50 mmol/l potassium phosphate buffer (pH 6.5) containing β-NADPH (15 μmol/l) and between 1.25 and 20 μmol/l of EDHB. Initial velocities were determined by measuring the change in absorbance of the pyridine nucleotide at 340 nm in a 1-ml volume.

### Analysis of cellular apoptosis

A total of 4×10^5^ cells were plated in 6-well plates and cultured overnight. The cells were treated with EDHB at the IC_50_ EDHB for 24, 48, or 72 h and then harvested. The cells were stained with Annexin-V and PI using a cellular apoptosis detection kit (Biosea Biotech., Beijing, China) according to the manufacture's procedure. The fluorescence intensity was detected by flow cytometry.

### Immunofluorescence

Cells growing in the logarithmic phase were seeded onto glass coverslips overnight, fixed with 4% (w/v) paraformaldehyde for 30 min, and rinsed with PBS for 15 min. The cells were subsequently permeabilized with 0.1% (w/v) Triton X-100 at RT for 10 min, rinsed with PBS, and incubated with PBS containing 2% BSA for 30 min. Next, the cells were incubated for 1 h at RT with the appropriate primary antibodies, rinsed several times, and incubated at RT for 30 min with the corresponding fluorescent secondary antibodies. Fluorescence images were captured with a Nikon ECLIPSE 80i microscope.

### siRNA transfection

Prior to transfection, cells were plated in 6-well plates and allowed to reach 80% confluence. After 12–16 h of culture, 5 μl of Lipofectamine 2000 and 5 μl of duplexed siRNA (sequences: 5′-UCCAGUGUCU GUAAAGCCATT-3′, 5′-UGGCUUUACAGACACUG GATT-3′, 5′-GGCCGUGGAGAAGUGUAAATT-3′, 5′-UUUACACUUCUCCACGGCCTT-3′, 5′-GGAGAUG AUCCUCAACAAGTT-3′, and 5′-CUUGUUGAGG AUCAUCUCCTT-3′; GenePharma, Shanghai, China) were separately pre-incubated in 150 μl of Opti-MEM for 5 min. These two solutions were then mixed and incubated at RT for 30 min. The cells were washed twice with PBS and pre-incubated in 700 μl of serum-free culture medium. The siRNA and liposome mixture was then added to the culture medium, and the medium was replaced with culture medium containing 20% FBS after 6 h. The cells were harvested after 48 h, and knockdown was verified by Western blot analysis.
